# 

**Published:** 1898-11

**Authors:** 


					﻿Vol. XVIII.
NOVEMBER; 1898
No. 11'
THE
HOMEOPATHIC pH^lL'lAJI,
A Monthly Journal of Homoeopathic Materia Medica
and Clinical Medicine.
“ He is a freeman whom truth makes free, and all are slaves beside."
“Seek the Truth: come whence it may, cost what it will."
EDITED AND PUBLISHED BY
WALTER M. JAMES, M. D.
PHILADELPHIA :
Ko. 1231 LOCUST STREET.
‘Yearly Subscription, $2.50, invasrialdy in adt/anee. Single numbers, 25 els. j
Entered at the Philadelphia Poat-Offloe as Second-Class Mail Matter.
				

